# Parliament and the Profession

**Published:** 1917-01-06

**Authors:** 


### Parliament and the Profession.

### Hospital Units.

Mr. Watt inquired of the Financial Secretary to the War Office as to the uses of the complete fully-trained hospital units in this country which are in readiness for active service. Mr. Macpherson explained that at the moment the Forces overseas have been fully supplied with medical units, and there is only occasion to send individuals as reinforcements.

### Royal Army Medical Corps Pay.

Mr. Forster admitted the suggestion in a question put by Sir C. Kinloch-Cooke that many temporary captains of the R.A.M.C. on active service are receiving higher pay than Regular captains, who in some cases are in command of the units to which such temporary officers are attached, but stated that the difference in total emoluments is almost negligible, and after full consideration it is not considered necessary to disturb the existing arrangements.

### Tuberculosis (in Cattle) Order.

Sir R. Winfrey explained to Mr. Rowntree that the temporary withdrawal of the Tuberculosis (in Cattle) Order would not have the effect of lessening the precautions against infected milk being sold for human consumption, which is fully restrained by Local Government Board Orders and by various local Acts. It is not proposed to bring the Order in question into operation again until after the war.

